# P25 Ceftazidime/avibactam and cefiderocol use in adults in a South London Trust

**DOI:** 10.1093/jacamr/dlad066.029

**Published:** 2023-06-14

**Authors:** Laura Kmentt, Rajeni Thangarajah, Balram Rathish, Aisling Brown

**Affiliations:** Department of Infectious Diseases, Guy’s & St Thomas’s NHS Foundation Trust, London, UK; Department of Infectious Diseases, Guy’s & St Thomas’s NHS Foundation Trust, London, UK; Department of Pharmacy, Guy’s & St Thomas’s NHS Foundation Trust, London, UK; Department of Infectious Diseases, Guy’s & St Thomas’s NHS Foundation Trust, London, UK; Department of Infectious Diseases, Guy’s & St Thomas’s NHS Foundation Trust, London, UK

## Abstract

**Background:**

NHS England now subsidizes pharmaceutical firms to invest in new antibiotic development. Currently, this applies to cefiderocol and ceftazidime/avibactam for treatment of patients with severe bacterial infections non-responsive to other antibiotics. The aims are to reduce the risk of resistance and protect the longevity of antimicrobials by restricting use to certain patients with sepsis, hospital/ventilator-associated pneumonia and bloodstream infections. Acute hospital trusts bear most of the drug cost but receive a 19%–33% discount.

**Methods:**

Pharmacy records for ceftazidime/avibactam or cefiderocol from July to December 2022 were accessed. Medical notes and laboratory systems were interrogated for clinical information as part of required Blueteq reporting.

**Results:**

15 prescriptions of cefiderocol and ceftazidime/avibactam were reviewed in 14 patients. The total number of days of therapy was 268 (112 + 156 for cefiderocol and ceftazidime/avibactam respectively). Median treatment duration was 11.5 (IQR 7–16.5) and 10 (IQR 5–22) days for cefiderocol and ceftazidime/avibactam, respectively. All infections were considered healthcare associated. Target organisms were Enterobacterales (39%), *Pseudomonas* spp. (28%), *Acinetobacter* spp. (11%) and *Stenotrophomonas* spp. (22%) (Figure 2). Twenty-one percent had polymicrobial infections and 50% of patients received adjunctive antibiotics (25% cefiderocol and 71% ceftazidime/avibactam). In-hospital mortality was 64%. All Enterobacterales, *Acinetobacter* and *Pseudomonas* isolates underwent phenotypic carbapenem susceptibility testing, and 100% of these were found to be resistant to at least one carbapenem. Where phenotypic carbapenem resistance was detected in Enterobacterales, reflex carbapenemase testing in accordance with laboratory protocol was performed for 71%, with a gene detected in 71% of these. For carbapenem-resistant *Acinetobacter* isolates, reflex ceftazidime/avibactam testing in accordance with laboratory protocol was performed in 100%. There is no local protocol in place for reflex cefiderocol/ceftazidime/avibactam susceptibility testing in multidrug-resistant *Pseudomonas* isolates. The median turnaround time from sample collection to result authorization was 2 (IQR 1.75–10) and 7 (IQR 2–9) days for cefiderocol and ceftazidime/avibactam, respectively. Use was empirical at the time of prescription in 40% of cases and appropriate (based on final AST results) in 80%. An infection specialist was involved in the initial prescription in all cases, and bedside review occurred in all cases.

**Conclusions:**

Use of high-cost antimicrobials is currently led by infection specialists in our Trust, often empirical initially but usually appropriate based on final results. Practices around reflex expanded susceptibility of clinically significant MDR Gram negatives, and carbapenemase testing of Enterobacterales should be improved. Optimizing these processes should be prioritized as the infection sciences laboratory moves to a central hub.

